# Gambling participation and policies in Malaysia

**DOI:** 10.1186/s40405-016-0012-1

**Published:** 2016-05-21

**Authors:** Jasmine M. Y. Loo, Kai Lit Phua

**Affiliations:** Monash University Malaysia, Bandar Sunway, Selangor Darul Ehsan Malaysia

## Abstract

Regulatory policies for responsible gambling practices in Asia are constantly evolving as the gambling industry and technological landscape change over time. Malaysia makes an interesting case study for a commentary on gambling participation and policies, as this country has a unique dual justice system with religious and ethnic diversity that may impact on the way in which gambling activities are regulated. This regulatory ecosystem has important consequences on behaviour change, treatment approaches and recovery processes involved in gambling disorder. This commentary will discuss evidence for Malaysian gambling antecedents, public policy and socioeconomic impacts of gambling, possible costs and benefits of gambling legalization, and issues pertinent to regulating gambling activities in Malaysia.

## Gambling definitions, prevalence and factors associated with gambling participation

Gambling disorder is classified under “Substance-related and addictive disorders” in the latest edition of the Diagnostic and Statistical Manual of Mental Disorders (DSM-5; American Psychiatric Association 2013). The new classification is a departure from the previous “Impulse control disorders” taxonomy in DSM-IV-TR that defined gambling disorder as “pathological gambling” stemming from an inability to control gambling impulses (American Psychiatric Association 2000). This significant change maintains consistency with the increasing empirical evidence to suggest that gambling behaviour stimulates the brain reward and feedback system similar to substance abuse (Aasved 2003; Bechara et al. 1994; Brewer and Potenza 2008). Furthermore, gambling disorder symptoms of dependence, craving, tolerance and withdrawal with high rates of relapse are comparable with substance use disorders (Ryan 2013; Skinner and Aubin 2010).

The diagnostic criteria for gambling disorder include elements of preoccupation and a inability to stop gambling that contributes to a substantial negative impact on the gambler’s personal life and significant others (American Psychiatric Association 2013). Commonly used terms to describe disordered gambling behaviour in the literature includes “compulsive gambling,” “problem gambling (PG),” and “pathological gambling” (Loo et al. 2008; Raylu and Oei 2002). Clinicians often use the definition “pathological,” while rehabilitation programs (e.g., Gamblers Anonymous worldwide) and laypersons use the term “compulsive” (Choong et al. 2014). The term “problem” is preferred as it evades the biomedical and deprecating implications of “pathological” (Walker and Dickerson 1996). Problem gambling primarily describes gambling behaviour at an earlier stage with a spectrum of negative consequences to self and significant others but may not necessarily fulfill the diagnostic criteria (Neil et al. 2005; Rosenthal 1989). In this article, terms such as gambling, problem gambling and gambling disorder will be used in the discussion on policies and regulation.

Prevalence of problem gambling in Malaysia was determined using the largest state of Selangor (population of 5.6 million; Department of Statistics Malaysia 2010) with a proportionate stratified random sampling method and Problem Gambling Severity Index as a community prevalence estimate (Ferris and Wynne 2001; Loo et al. 2011). In this study, 4.4 % of the general Malaysian population were categorized as problem gamblers while 10.2 % were moderate-risk gamblers (Loo and Ang 2013). This prediction signifies that approximately 246,400 Malaysians in Selangor are potentially problem gamblers while 571,200 Malaysians in Selangor are moderate-risk problem gamblers.

Although state-specific, the results suggest that Malaysians are participating in gambling activities and the prevalence rates are comparatively on the higher end of the spectrum—i.e., 4.4 % (Loo and Ang 2013)—compared to other Asian populations, which report problem gambling rates ranging from 1.4 to 2.5 % (Blaszczynski et al. 1998; Fong and Ozorio 2005; Winslow et al. 2015). More support is needed in the advancement of research, treatment and policies to protect potentially vulnerable Malaysian gamblers and their families. Reports from treatment service providers often articulate the need to conduct prevention and awareness programs in the community to assist in the recovery process of treatment-seeking gamblers and also provide educational support to family members among communities where recreational gambling may be culturally acceptable (Choong et al. 2014; Loft and Loo 2014). Accessibility of online casinos via smartphones and tablet applications have increased the ease of access to gambling venues (Gainsbury 2015) and ultimately problem gambling in this nation.

As shown in Table 1, factors associated with gambling in Malaysia on socio-demographic factors associated with participation in gambling can be determined by analysing household expenditures (Tan et al. 2010). Factors identified to be associated with higher gambling expenditures were being of Chinese ethnicity, lower education levels, higher income, and paternal-headed or non-white collar households. Several studies attributed higher gambling participation to rigorous marketing strategies implemented to promote gaming operators and gambling venues in a positive light (Yoong et al. 2013). Similarly, customers with relativist ethical ideologies placed more perceived value on gambling and were more committed to gambling participation (Ndubisi et al. 2012).

**Table 1:** Empirical studies conducted in relation to gambling among Malaysians

Authors/Title	Design	Participants	Findings
Tan et al. (2010) “Socio-demographic determinants of gambling participation and expenditures: Evidence from Malaysia”	Analysed pre-existing data from the 2004–2005 Malaysian Household Expenditure Survey from Malaysian Department of Statistics Stratified multistage with area probability sampling method Examined total monthly expenditures on gambling activities	*N* = 6117 non-Muslim households (3200 Chinese, 810 Indians, 2107 other ethnicities) with an average income of RM3065	Socio-demographic factors associated with higher level of gambling expenditures are Chinese, affluent, paternal-headed, younger and non-white collar households Education and age affect gambling expenditures among Chinese households Higher income and paternal households predicted higher gambling expenditures White-collar Chinese and Indian households have lower gambling probabilities
Yoong et al. (2013) “This is not gambling but gaming: Methods of promoting a lottery gaming company in a Malaysian daily”	Critical discourse analysis of articles published in a Malaysian newspaper on a lottery gaming company Qualitative research—Fairclough’s framework of analysis	Not applicable (used published materials as data)	Charity activities used as false positive associations with lottery company’s gambling activities Journalistic and reporting integrity of the local daily are questionable Endorsements by influential individuals as a positive association Winnings published regularly can impact the perception of a high probability of striking a win through lottery, which can be misleading to readers
Ndubisi et al. (2012) “Ethical ideologies, perceived gambling value, and gambling commitment: An Asian perspective”	Relationship marketing paradigm Field survey of customers in a leading Asian gambling establishment over 3 weeks Evaluated inter-relationships between ethical ideology, gambling commitment and perceived gambling value	*N* = 382 customers (50.8 % males, 49.2 % females; 80 % Chinese, 20 % non-Chinese; 2.1 % Muslims, 97.9 % Muslims; 41.6 % low income, 59.4 % middle to high income)	Patrons with relativist ethical ideologies were more committed to gambling as compared to idealist ideologies Negative association between idealism and perceived gambling value Positive association between relativism and perceived gambling value Perceived gambling value moderates: (1) the negative association between idealism and gambling commitment, and (2) the positive association between relativism and commitment
Sawari et al. (2010) “On the question of gambling in giving prizes to the holders of savings certificates: An Islamic analysis on Malaysian practice”	Qualitative research—content analysis of Islamic *Syari’ah* literature and interviews Investigated the Islamic legality of prize-giving practices through monthly draws to selected holders of a savings account in a National Savings Bank of Malaysia	Not specified in the article	In *Syari’ah* law, gambling and retaining revenues gained through any form of gambling is prohibited Personal savings certificate (PSC) is not associated with gambling under *Syari’ah* due to the winning and losing payout ratio
Tan et al. (2009) “The demand for vices in Malaysia: An ethnic comparison using household expenditure data”	Analysed pre-existing data from the 2004–2005 Malaysian Household Expenditure Survey Econometric estimation of a trivariate Tobit system to examine the demand for vices (tobacco, alcohol and gambling)	*N* = 14,082 households (37.57 % reported tobacco expenditures, 7.87 % alcohol, 7.46 % gambling expenditures)	Higher education predicted lower expenditures on vices White-collar headed households are less likely to smoke Higher disposable income predicted higher tendency to spend on tobacco (Malay households) and all vices (Chinese and all other ethnicities) Male-headed households spend more on vices as compared to female-headed households
Tudin and Woon (2012) “Factors influencing individuals’ gambling behaviour: A case study in Malaysia”	Convenience sampling survey of customers in Casino de Genting, Malaysia Examined demographics, gambling behaviour and factors contributing to gambling decisions (i.e., marketing activities, sociocultural environment and psychological variables)	*N* = 200 participants above 19 years (71.5 % Malaysians, 52.5 % male)	Marketing activities (promotions, services, positioning and winnings) predicted higher gambling behaviour In this study, psychological variables (motivation, personality, perception, cognitions) did not significantly predict gambling behaviour. This result can be attributed to small sample size and weaknesses in data analysis methods.
Choong et al. (2014) “The experience of recovering gamblers in Malaysia: A phenomenological study”	Qualitative analyses on interviews conducted with treatment seeking gamblers	*N* = 10 (phenomenological analysis on recovering gamblers)	Family support is essential in the recovery from gambling disorder A reduction in enabling behaviours (e.g., helping gambler to pay off debts) is a key turning point for gambling recovery
Loft and Loo (2014) “Understanding the Mechanisms Underlying Gambling Behaviour and Sleep”	Quantitative analyses on sleep difficulty, sleep habits, arousability, self-regulatory capacity and problem gambling severity	*N* = 59 (treatment seeking gamblers)	Self-regulatory capacity mediates the relationship between problem gambling and sleep difficulty Self-regulatory capacity is also a significant mediator between problem gambling and negative sleep habits Arousability predicted sleep difficulty and negative sleep habits

Nevertheless population surveys, household expenditure studies and surveys on casino customers have differing research questions and parameters, which may bias the data towards these factors and hold limitations with how the data is collected. Future investigations would benefit from varied sources and population to enable data triangulation. All studies presented in Table 1 were predominantly conducted in business, finance or psychology fields; and were methodologically sound with findings that build a foundation for future research. Further investigations are needed in varied topics such as illegal gambling antecedents, preferred gambling activities, risk factors, social responsibility programs practiced by industry stakeholders, effective prevention and treatment programs, etc.

In a study geared toward marketing perspectives, Ndubisi et al. (2012) suggested that gaming operators should improve their corporate image through progressive advertisements of corporate social responsibility (CSR) initiatives within the organization. Yet suggestions are likely to vary according to the perspectives or conventions from which these researchers come from (e.g., consumer, community, governmental stakeholders, gambling industry). Corporate social responsibility when viewed from the standpoint of the gambling industry may suggest initiatives that provide beneficial outcomes to the public image and eventual revenues for the gaming company. Meanwhile, CSR from a governmental standpoint may include regulatory policies and safeguards that protect the interests of the country and population. It would be beneficial, however, to work towards mutual co-operation between stakeholders (community, government and industry) and minimize doubling up of efforts for the same issue. This can be conducted with selected initiatives that allow information sharing and would potentially minimize wastage of resources spent by multiple stakeholders.

The influence of the local media has been questioned (Yoong et al. 2013). For example, certain lottery establishments were portrayed positively through endorsements by influential individuals, creation of positive associations with charities, and publications of regular winnings that creates a perception of high winning probability. Odds of winning and house advantage was not published consistently in a transparent manner where such transparency should be common practice among proponents of responsible gambling (World Lottery Association 2013). Policies and reliable regulatory enforcements are essential in this region and nation to protect vulnerable at-risk problem gamblers by ensuring that gaming operators adhere to responsible gambling practices and social responsibility safeguards. Gambling policy is under-developed in Malaysia and is in need for further development through discussions with gambling or gaming stakeholders. These stakeholders may include government regulators, gaming industry, treatment providers and research institutes. Adopting a public health perspective in addressing these issues is potentially a worthwhile initiative.

Regulating gambling policies in multi-ethnic and culturally diverse nations can be controversial, political and should be treated with caution. Malaysia is unique in its regulations as a nation with a separate Islamic *Syari’ah* law enforced for Muslim individuals. Gambling is illegal and punishable under the Islamic *Syari’ah* law if a Muslim devotee is implicated in the activity or has harboured money gained through gambling activities. Furthermore, Muslim charity organizations would never accept donations from organizations that profit through gambling revenues, as such monies are considered “unclean.” Adherence to the Islamic law transcends most aspects of life as a Muslim and audits are conducted to ensure compliance.

Historically, banking and finance studies were conducted to examine compliance of the reward system of personal savings certificate to the Islamic *Syari’ah* law (Sawari et al. 2010; Please see Table 1). Muslim individuals are somewhat “protected” from problem gambling as they are not legally allowed to enter gambling venues. Nevertheless, due to social acceptance of gambling among certain ethnicities (i.e., Chinese and Indians) may not be protected by governmental authorities that are predominantly Muslim who may be hesitant in devising stringent gambling regulations to ensure responsible gambling practices for fear of stirring religious disharmony. Despite these sentiments, it is fundamental for the development of the nation and protection of its citizens at large (i.e., other ethnic minorities) that responsible gambling policies are implemented and regulated ethically with transparent protocols. This should be done through consultations with key stakeholders (governmental authorities, gaming operators and treatment providers) and academic specialists in this area.

## Gambling history, public policy and socioeconomic impacts

Government policies often affects the growth and proliferation of the legalized gambling sector and the number, location, and size of gambling establishments allowed (Kearney 2005). When governments remove legal restrictions and encourage the proliferation of certain forms of gambling such as lotteries and casino gambling it may result in rapid growth of these forms worldwide (Richard 2010). In Malaysia, the first and only legal casino, the Casino de Genting, was opened at Genting Highlands resort in the state of Pahang in the early 1970 s. In spite of its sizable non-Muslim population, no further casinos have been allowed by the authorities to be opened in Malaysia partly because of the influence of Islam and its increasing impact on public policy in this country. There may be other factors influencing this process such as political reasons and business decision-making outcomes, which should be investigated in future research studies.

Previously, a social welfare lottery system was established by the Malaysian government, but it was subsequently terminated through the closure of its Social and Welfare Services Lotteries Board in 1991 (Commissioner of Law Revision Malaysia 1991). At present, in addition to the Casino de Genting, other legalized gambling entities include privately owned lottery operators such as Magnum Berhad, Sports Toto and Damacai. The non-casino gambling industry in Malaysia has been estimated to be worth US$2.99 billion (Berthelsen 2013). The dual legal system (*Syari’ah* and British Common System) present in Malaysian context allows for a unique influence on the gambling habits of its people. As approximately 60 % of the country’s population are Muslim individuals who abide by Islamic legalities and governmental positions are predominantly held by Muslims, there are considerable effects of Islam on public policy initiatives and decision making processes.

There appears to be an intensification of the trend of Islamisation in Malaysia. This is indicated by the increasing impact of Islam on public policy (e.g. in the area of education) and in popular discourse—including debates on how to deal with social problems such as gambling addiction. As an example of the latter, in year 2010, the highly influential non-governmental organisation *Angkatan Belia Islam Malaysia* or ABIM (Malaysian Muslim Youth Movement) called upon Malaysian Muslims to avoid indulging in any form of gambling as it allegedly destroys individual character, erodes the integrity of the family as a social institution and also has other serious negative effects on the socio-economic system (Malaysian Insider 2010). ABIM claimed that gambling is forbidden in Islam. It should be noted that Muslims in Malaysia have been arrested for gambling by state Religious Affairs Department officers under *Syari’ah* law based on media publications. Future research studies should be conducted to validate these claims and tabulate actual percentages.

## Gambling legalization

Governments can decide to legalize gambling for economic reasons such as increasing state revenues through entertainment taxes (Barmaki and Zangeneh 2009; Hancock et al. 2008). A complete ban on gambling within a political jurisdiction is harder to enforce and may drive such activities underground, creating opportunities for organized crime groups to engage in illegal gambling operations, or induce gamblers to cross state or national borders to gamble. “Underground gambling” or illegal betting include gambling activities carried out in unlicensed premises, unregulated lotteries and illegal bookmaking operations.

When a political jurisdiction forbids or restricts gambling activities, gamblers may be able to overcome this by traveling to another nearby area where gambling is legal and easily accessible. For example, Native American reservations in the USA are semi-autonomous where indigenous authorities have federally-recognised power to allow the establishment of casinos and other gambling establishments (Romboy 2013). Another example would be individuals who travel from the socially conservative state of Utah, where all forms of gambling are banned, to neighbouring Idaho to buy lottery tickets (Romboy 2013) or to Nevada and its plentiful casinos in cities such as Las Vegas and Reno. In Malaysia, the equivalent would be individuals travelling from the socially conservative state of Kelantan to gamble in the casino located in the Genting Highlands in Pahang state. The growth and availability of internet gambling options may inevitably increase accessibility and gambling participation rates.

Gambling is illegal in Malaysia for the wider population unless a license or permit has been granted by the authorities such as the *Unit Kawalan Perjudian* (Betting Control Unit) of the Ministry of Finance. Thus, it is illegal to gamble in public places or even at home. It is also illegal to engage in bookmaking or to operate a lottery without a license or permit. However, this does not mean that illegal gambling activities are not rampant. Malaysian media, for example, highlight an increase in illegal venues, bookmaking and gambling activities that have been identified by law enforcement officers. Furthermore, gambling machines such as slot machines, jackpot machines, and “turfking” machines are available even if they have been prohibited from being placed in venues not licensed for gambling (The Star Online 2006). Further empirical investigations in gambling research is needed to examine actual percentages and extent of incidences reported in media and police reports.

Further, while contests and competitions are also not permitted by the Ministry of Finance in Malaysia (Kuek 2011), their set-up promotes participation in the following: (1) Participants answer questions and their entries are randomly selected. The winning entry is the one with all correct answers. In the event of a tie, the winning entry is determined by drawing lots; (2) Members of the public fill in forms. Forms picked by drawing lots are awarded prizes; (3) Participants predict the outcome of a competition; and (4) Participants guess the number of items in a container or the weight of a display item.

In these competitions, prosecution may not occur since compliance is the responsibility of the individual business establishments and self-governance is expected. This is an illustration of legalities in Malaysia whereby legislation may occasionally lack enforcements, which eventually results in non-compliance by the public. Policy is also lagging in that the Social and Welfare Services Lotteries Board Act 1950–1962, the Lotteries Act 1953 and the Gaming Tax Act 1972 are dated and in need of a thorough review.

## Benefits of legalized gambling

The legalization of gambling can be a contentious issue (Dunstan 1997). For example, the legalization of sports betting by the administration of Prime Minister’s office in 2010 ahead of the World Cup soccer tournament resulted in controversial speculations in Malaysia. The political opposition criticised the government for awarding the exclusive license to Berjaya Group’s Ascot Sports *Sendirian Berhad* (Ascot Sports Private Limited) without an open tender, which should be the standard procedure (Malaysian Insider 2010).

Some have argued that there are benefits arising from gambling legalization (Zheng and Hung 2012). Besides the utility and satisfaction gained by gambling product consumers without violating legal norms, there are allegedly economic benefits for the society at large. These include the creation of jobs through legalized gambling venues (Richard 2010), taxation revenues gained by the government (Shaffer and Korn 2002), and socioeconomic development of the community (Zheng and Hung 2012). For example, growth of remote cities such as Las Vegas in Nevada, USA and Macau, China (Dunstan 1997) whereby there are infrastructure improvements and constant influx of tourism revenues.

Revenues collected by other governments from gambling include taxes levied on gambling establishments, nett funds derived from state lotteries (i.e., after deducting for prize money given out, administrative costs, and advertising costs), and revenue from licenses and other fees (Kearney 2005). In Malaysia, socioeconomic development has been promoted with the opening of a legal casino at Genting Highlands in Pahang. It has been accompanied by a proliferation of hotels, restaurants, shopping malls and theme parks that attract tourism and associated revenues from locals and neighbouring countries such as China, Thailand, Indonesia, and Singapore. Future investigations need to be conducted to ascertain the extent of socioeconomic impact.

Shaffer and Korn (2002) stated that recreational gambling can have positive effects on mental health by providing a sense of connectedness and socialization through self-regulated leisure time entertainment. Some forms of gambling (such as card games and mah-jong) encourage social interactions as it requires multi-player participation. Certain forms of gambling can benefit individuals by enhancing concentration and memory, promoting problem-solving skills and mathematical proficiency, as well as stimulating eye-hand coordination.

## Disadvantages of legalized gambling

In view of the potential benefits of legalized gambling, there are also detrimental impacts of both legal and illegal gambling. Health expenditures include and are not limited to psychological treatment costs for gambling disorder, medical prescriptions for gamblers and family members, family therapy, and emergency treatment for suicidal attempts stemming from inability to cope with heavy gambling debts (Australian Medical Association 2013; Raylu et al. 2008; Shaffer and Korn 2002). There are also associations found between problem gambling and chronic physical conditions (Black et al. 2013; Pilver and Potenza 2013).

Problem gamblers often exhibit high levels of comorbid mental health disorders and engage in substance abuse (Krmpotich et al. 2015). All these would contribute to healthcare costs at individual and societal levels (Lorains et al. 2011). Other financial costs to the gambler and family members (Kearney 2005) include debts arising from problem gambling, personal bankruptcies, disruption or loss of employment, reduced household savings, poverty, and reduced household spending on other essential goods and services. Reduced spending by households on non-gambling related goods and services would affect other sectors of the economy such as other leisure and entertainment services. Another potential economic cost would be reduced productivity at work if the employee is distracted by gambling-related activities, which will in turn affect the organization’s productivity.

According to Dunstan (1997), the opening of casinos and integrated resorts in Atlantic City was accompanied by a significant decrease in the number of restaurants throughout the city. Thus, when the state of Louisiana legalized a casino, casinos were prohibited from having in-house hotel facilities and restaurants. It is evident that the growth of the gambling industry may be accompanied by the reduction in economic selling power of other smaller businesses in the community. This may produce an undesired effect of slower economic growth of the community at large.

Legalized gambling, although it decriminalizes certain forms of gambling, can also lead to other types of crime such as theft, money laundering and other financial crimes to obtain money for gambling activities or to pay gambling debts. In Malaysia, there is the illegal loan sharks phenomenon (i.e., “*Ah Longs*”) where the gambler and family members are potentially harassed for repayment of loans given out at usurious and unreasonable interest rates. The increased cost of policing would be a disadvantage in terms of the debate over gambling legalization. All governmental decisions and policies should weigh both benefits and disadvantages of gambling legalization, while engaging with the main stakeholders to ensure that foundations and structures are in place to accommodate the economic and societal impacts of the gambling industry on a nation.

## General discussion

This commentary iterates that governmental policies can alter the size and form of the legalized gambling sector. These policies are partly responsible for the spread of casino gambling in some nations (Richard 2010). In the USA, Native American tribal councils have the right to set up and operate gambling establishments on their reservations, subject to the Indian Gaming Regulatory Act of 1988 (Cornell 2008). Shaffer and Korn (2002) mentioned that gambling aboard cruise ships and riverboats have appeared either to circumvent gambling laws or to comply with legal restrictions.

An empirical study carried out on patrons of Casino de Genting, Malaysia (Rabaah and Woon 2012) found that marketing activities can affect gambling participation. The appearance and growth of newer forms of gambling may be at the expense of existing forms of gambling (Dunstan 1997). This type of change has been called “cannibalization.” Thus, the appearance of “Interactive Gambling” via the Internet, smart phones and digital TV (Australian Medical Association 2013) could conceivably lead to cannibalization by reducing participation in other forms of gambling, especially those which require the physical presence of the gambler. Internet gambling poses a significant challenge to regulatory authorities (Gainsbury and Wood 2011), as such new forms of gambling may affect public revenues derived from legalized gambling if interactive gambling is largely non-regulated and insufficiently taxed.

Findings from other countries have highlighted the inconsistency of government policies with regard to gambling. For example, Macao relies on tax revenues derived from casinos to deal with gambling-related problems (Gu and Tam 2011). Meanwhile in Australia, state governments have liberalized gambling policy though at the same time, revising regulations and coming up with new ones to deal with negative socio-economic impact of growth in the gambling industry (Delfabbro and King 2012). In Malaysia, the government has terminated its social welfare lottery but business is thriving at gambling establishments operated by privately-owned lottery organizations.

Researchers have pointed out that some demographic groups are more vulnerable and have a higher risk of developing gambling problems. Groups at higher risk include individuals of Chinese ethnicity (Loo et al. 2008; Yen and Wu 2013), minorities such as Indigenous populations (Breen and Gainsbury 2013; Dyall 2010), the poor and less educated (Clotfelter and Cook 1989), the elderly (McNeilly and Burke 2000), and youth (Barmaki and Zangeneh 2009). It has also been noted that people with pre-existing mental health conditions are at a higher risk of developing problem gambling (Rodda et al. 2012).

Since Malaysia has a large population sub-group of Chinese (approximately 25 % of the total population) and Indian ancestry (approximately 12 %), this has important policy implications for this nation. More efforts in the area of problem gambling prevention would need to be targeted at the non-Muslim Malaysian community who are legally allowed and culturally acceptable to gamble in Malaysia, including its more affluent members and at-risk groups since they have been found to spend more on gambling (Tan et al. 2009, 2010).

In the case of the poor and less educated, participation in forms of gambling such as state lotteries would actually be a form of regressive taxation (Clotfelter and Cook 1989) since profits derived from such lotteries go into government resources. An analysis of 13 countries (Richard 2010) with respect to legalization of casino gambling resulted in the suggestion that religiosity, as measured by frequency of church attendance, was a significant barrier to casino legalization. No additional casino has been allowed to open in Malaysia since the launching of the Casino de Genting in the early 1970s. Neighbouring country, Singapore’s decision to legalize casino gambling with the motivation of promoting “integrated resorts” to increase tourism revenues was opposed by religious groups and also other community groups but the government went ahead nonetheless for economic and tourism reasons (Ministry of Trade and Industry of Singapore 2005).

There are many challenges ahead (e.g., dual legal system, democracy in multi-ethnic/religious society, cultural tolerance and sensitivity, low political motivation) for effective regulation of gambling in Malaysia and countries with multi-ethnic communities that have similar demographics. All stakeholders in responsible gambling initiatives play an important role in managing the progression of effective regulatory policies that would protect consumers through appropriate treatment provision (Raylu et al. 2013) and optimize compliance on responsible gambling policies from gambling operators. Theoretical and translational research studies precede successful implementation and adherence to regulatory policies and initiatives that ultimately aim to minimize harm associated with disordered gambling. Successful implementations of regulatory policies require the compliance of the gambling industry and community support. Much research is needed in this area to inform effective governmental policy making decisions that benefit the population at large.

## References

[CR1] Aasved M (2003). The biology of gambling: The gambling theory and research series.

[CR2] American Psychiatric Association (2000). Diagnostic and statistical manual of mental disorders: DSM-IV-TR.

[CR3] American Psychiatric Association (2013). Diagnostic and statistical manual of mental disorders: DSM-5.

[CR4] Australian Medical Association. (2013). *Health effects of gambling*. Retrieved from https://ama.com.au/position-statement/health-effects-problem-gambling

[CR5] Barmaki R, Zangeneh M (2009). Canadian dream, capitalism, and the state: Structural conditions of youth gambling in Canada. International Journal of Mental Health and Addiction.

[CR6] Bechara A, Damasio AR, Damasio H, Anderson SW (1994). Insensitivity to future consequences following damage to human prefrontal cortex. Cognition.

[CR7] Berthelsen, J. (2013). Threat to Malaysia’s gaming tables? *Asian Sentinel, May 1.* Retrieved from http://www.asiasentinel.com/index.php?option=com_content&task=view&id=5378&Itemid=229

[CR8] Black DW, Shaw M, McCormick B, Allen J (2013). Pathological gambling: relationship to obesity, self-reported chronic medical conditions, poor lifestyle choices, and impaired quality of life. Comprehensive Psychiatry.

[CR9] Blaszczynski A, Huynh S, Dumlao VJ, Farrell E (1998). Problem gambling within a Chinese speaking community. Journal of Gambling Studies.

[CR10] Breen H, Gainsbury S (2013). Aboriginal gambling and problem gambling: A review. International Journal of Mental Health and Addiction.

[CR11] Brewer JA, Potenza MN (2008). The neurobiology and genetics of impulse control disorders: Relationships to drug addictions. Biochemical Pharmacology.

[CR12] Choong LL, Loo JMY, Ng WS (2014). The experience of recovering gamblers in Malaysia: A phenomenological study. Asian Journal of Gambling Issues and Public Health.

[CR13] Clotfelter CT, Cook PJ (1989). Selling hope: State lotteries in America.

[CR14] Commissioner of Law Revision Malaysia. (1991). *Social and Welfare Services Lotteries Board (Dissolution) Act 1991*. Retrieved from http://www.agc.gov.my/Akta/Vol.10/Act470.pdf

[CR15] Cornell S (2008). The political economy of American Indian gaming. Annual Review of Law and Social Science.

[CR16] Delfabbro P, King D (2012). Gambling in Australia: Experiences, problems, research and policy. Addiction.

[CR17] Department of Statistics Malaysia. (2010). *Population distribution and basic demographic characteristic report*. Retrieved from Kuala Lumpur, Malaysia: http://www.statistics.gov.my/portal/index.php?option=com_content&id=1215

[CR18] Dunstan R (1997). Gambling in California.

[CR19] Dyall L (2010). Gambling: A poison chalice for indigenous peoples. International Journal of Mental Health and Addiction.

[CR20] Ferris, J., & Wynne, H. (2001). *The Canadian problem gambling index: Final report*. Retrieved from Ottawa (ON).

[CR21] Fong DK, Ozorio B (2005). Gambling participation and prevalence estimates of pathological gambling in a far-east city: Macao. UNLV Gaming Research & Review Journal.

[CR22] Gainsbury SM (2015). Online gambling addiction: The relationship between internet gambling and disordered gambling. Current Addiction Reports.

[CR23] Gainsbury SM, Wood R (2011). Internet gambling policy in critical comparative perspective: The effectiveness of existing regulatory frameworks. International Gambling Studies.

[CR24] Gu X, Tam PS (2011). Casino taxation in Macao: An economic perspective. Journal of Gambling Studies.

[CR25] Hancock L, Schellinck T, Schrans T (2008). Gambling and corporate social responsibility (CSR): Re-defining industry and state roles on duty of care, host responsibility and risk management. Policy and Society.

[CR26] Kearney MS (2005). The economic winners and losers of legalized gambling. National Tax Journal.

[CR27] Krmpotich T, Mikulich-Gilbertson S, Sakai J, Thompson L, Banich MT, Tanabe J (2015). Impaired decision-making, higher impulsivity, and drug severity in substance dependence and pathological gambling. Journal of Addiction Medicine.

[CR28] Kuek, P. Y. (2011). *And the winner is….*Retrieved from http://www.skrine.com/and-the-winner-is

[CR29] Loft MH, Loo JMY (2014). Understanding the mechanisms underlying gambling behaviour and sleep. Journal of Gambling Studies.

[CR30] Loo JMY, Ang KT (2013). Prevalence of problem gambling in Selangor urban areas.

[CR31] Loo JMY, Oei TPS, Raylu N (2011). Psychometric evaluation of the problem gambling severity index-Chinese version (PGSI-C). Journal of Gambling Studies.

[CR32] Loo JMY, Raylu N, Oei TPS (2008). Gambling among the Chinese: A comprehensive review. Clinical Psychology Review.

[CR33] Lorains FK, Cowlishaw S, Thomas SA (2011). Prevalence of comorbid disorders in problem and pathological gambling: systematic review and meta-analysis of population surveys. Addiction.

[CR34] Malaysian Insider. (2010). Abim asks reasons for sports betting licence. *Malaysian Insider, May 24*. Retrieved from http://www.themalaysianinsider.com/malaysia/article/abim-asks-reasons-for-sports-betting-licence

[CR35] McNeilly DP, Burke WJ (2000). Late life gambling: The attitudes and behaviors of older adults. Journal of Gambling Studies.

[CR36] Ministry of Trade and Industry of Singapore. (2005). *Statement by Prime Minister Lee Hsien Loong on Integrated Resort: Proposal to develop integrated resorts*. Retrieved from http://www.mti.gov.sg/MTIInsights/Documents/MinisterialStatement-PM18apr05.pdf.

[CR37] Ndubisi NO, Nataraajan R, Chew J (2012). Ethical ideologies, perceived gambling value, and gambling commitment: An Asian perspective. Journal of Business Research.

[CR38] Neil, P., Delfabbro, P., & O’Neil, M. (2005). *Problem gambling and harm: Towards a national definition*. Retrieved from Melbourne.

[CR39] Pilver CE, Potenza MN (2013). Increased incidence of cardiovascular conditions among older adults with pathological gambling features in a prospective study. Journal of Addiction Medicine.

[CR40] Rabaah, T., & Woon, C. Y. (2012). *Factors influencing individuals’ gambling behaviour: A case study in Malaysia*. FEB Working Papers Series, Number 2012. Universiti Malaysia Sarawak (UNIMAS). Kota Samarahan. Retrieved from http://www.feb.unimas.my/images/febwp/wps1202.pdf

[CR41] Raylu N, Loo JMY, Oei TPS (2013). Treatment of gambling problems in Asia: Comprehensive review and implications for Asian problem gamblers. Journal of Cognitive Psychotherapy.

[CR42] Raylu N, Oei TPS (2002). Pathological gambling: A comprehensive review. Clinical Psychology Review.

[CR43] Raylu N, Oei TPS, Loo JMY (2008). The current status and future direction of self-help treatments for problem gamblers. Clinical Psychology Review.

[CR44] Richard B (2010). Diffusion of an economic development policy innovation: Explaining the international spread of casino gambling. Journal of Gambling Studies.

[CR45] Rodda S, Lubman DI, Latage K (2012). Problem gambling: Aetiology, identification and management. Australian Family Physician.

[CR46] Romboy, D. (2013). Utahns find ways to gamble despite it being illegal in the state—but the cost is high. *Deseret News*. Retrieved from http://www.deseretnews.com/article/865582732/No-casino-no-lottery-yet-gambling-pervasive-in-Utah.html?pg=all

[CR47] Rosenthal RJ, Shaffer HJ, Stein SA, Gambino B, Cummings TN (1989). Pathological gambling and problem gambling: Problems of definition and diagnosis. Compulsive gambling: Theory, research, and practice.

[CR48] Ryan F (2013). Cognitive therapy for addiction: Motivation and change.

[CR49] Sawari MFM, Hassan R, Abdullah MF (2010). On the question of gambling in giving prizes to the holders of savings certificates: An Islamic analysis on Malaysian practice. Interdisciplinary Journal of Contemporary Research in Business.

[CR50] Shaffer HJ, Korn DA (2002). Gambling and related mental disorders: A public health analysis. Annual Review of Public Health.

[CR51] Skinner MD, Aubin H-J (2010). Craving’s place in addiction theory: Contributions of the major models. Neuroscience and Biobehavioral Reviews.

[CR52] Tan AKG, Yen ST, Nayga RM (2009). The demand for vices in Malaysia: An ethnic comparison using household expenditure data. Atlantic Economic Journal.

[CR53] Tan AKG, Yen ST, Nayga RM (2010). Socio-demographic determinants of gambling participation and expenditures: Evidence from Malaysia. International Journal of Consumer Studies.

[CR54] The Star Online. (2006). The adverse effects of gambling. *The Star Online, March 11*. Retrieved from http://thestar.com.my/fightcrime/resources/story.asp?file=/2006/3/11/resources/20060316165242&sec=resources

[CR55] Tudin, R., & Woon, C. Y. (2012). Factors influencing individuals’ gambling behaviour: A case study in Malaysia. *FEB Working Paper Series*(1202).

[CR56] Walker MB, Dickerson MG (1996). The prevalence of problem and pathological gambling: A critical analysis. Journal of Gambling Studies.

[CR57] Winslow M, Cheok C, Subramaniam M (2015). Gambling in Singapore: An overview of history, research, treatment and policy. Addiction.

[CR58] World Lottery Association. (2013). *Responsible Gaming Framework*. Retrieved from http://www.world-lotteries.org/cms/index.php?option=com_content&view=article&id=393&Itemid=100281

[CR59] Yen CF, Wu HYJ (2013). Gambling in Taiwan: Problems, research and policy. Addiction.

[CR60] Yoong D, Tan HK, Ng CM (2013). ‘This is not gambling but gaming’: Methods of promoting a lottery gaming company in a Malaysian daily. Discourse & Society.

[CR61] Zheng V, Hung EPW (2012). Evaluating the economic impact of casino liberalization in Macao. Journal of Gambling Studies.

